# Vastus Lateralis Transfer for Reconstruction of Hip Abduction following Superior Gluteal Nerve Injury

**DOI:** 10.1097/GOX.0000000000006123

**Published:** 2024-09-03

**Authors:** Vanessa Schuster, Henrik Lauer, Helene Hurth, Johannes C. Heinzel, Steven C. Herath, Adrien Daigeler, Jonas Kolbenschlag

**Affiliations:** From the * Department of Hand-, Plastic, Reconstructive and Burn Surgery, BG Klinik Tuebingen, University of Tuebingen, Tuebingen, Germany; † Department of Neurosurgery, University Hospital Tuebingen, University of Tuebingen, Tuebingen, Germany; ‡ Department of Trauma and Reconstructive Surgery, BG Klinik Tuebingen, University of Tuebingen, Tuebingen, Germany.

## Abstract

A 71-year-old man who had hip abductor insufficiency due to a chronic injury to the right superior gluteal nerve injury after lipoma resection presented to our outpatient clinic 1.5 years postoperatively with persistent pain, atrophy of the gluteus medius muscle, and Trendelenburg sign with a corresponding limp. A magnetic resonance imaging scan and neurophysiological diagnostics confirmed a chronic lesion of the superior gluteal nerve with completed reinnervation and absent pathological spontaneous activity, excluding neurosurgical options to restore hip abduction. Following interdisciplinary evaluation of the patient’s case, we performed a vastus lateralis transfer in May 2023 to stabilize the right hip joint. The entire vastus lateralis muscle was carefully freed from the surrounding tissue, and its attachment to the quadriceps tendon was separated. The proximal section of the lateral vastus was then fixed to the ilium and greater trochanter, while the muscle’s distal portion was sutured to a more proximal part of the quadriceps muscle. The postoperative course was without any complications, and the patient left the hospital with an abduction splint 10 days after surgery. When he presented to our outpatient clinic 10 weeks after surgery, he reported a significantly improved gait and reduction of pain. Trendelenburg sign was now absent, but right knee function was not impaired and the patient was able to ambulate without the regular need for an orthosis. A transfer of the vastus lateralis muscle is therefore a valuable option to restore hip abductor function in cases of chronic nerve lesions which exclude neurosurgical options.

Impairment of hip abduction, a movement mainly controlled by the gluteus medius muscle,^1^ is a problem after orthopedic procedures like total hip arthroplasty or tumor surgery.^2^ Besides direct injury to the muscle, which can be addressed by prompt surgical repair,^3^ such as refixation of the muscle to the proximal femur or the iliotibial band,^4^ an injury to the superior gluteal nerve can cause paralysis of the gluteus medius muscle.^5^ Such injuries can be addressed by direct nerve repair or nerve transfer surgery.^6^ However, neurogenic muscle degeneration due to chronic nerve injury excludes nerve surgery, given the inability to achieve meaningful motor recovery following denervation periods that exceed 12–18 months.^7,8^ Transferring an innervated muscle as a substitute may provide more functional gain.^3^ We report a case in which a transfer of the vastus lateralis muscle resulted in significant functional improvement of hip abduction in a patient with a chronic injury of the right superior gluteal nerve.

## CASE REPORT

We describe the case of a 71-year-old man who presented with insufficiency of the gluteus medius muscle due to a chronic lesion of the right superior gluteal nerve. He underwent lipoma resection in October 2021 in an external hospital and was discharged after 5 days without complications. During a visit 12 days later in the external hospital’s outpatient clinic, persistent hip abductor insufficiency was apparent, for which the patient was prescribed physical therapy. Six months postoperatively, he presented in the outpatient clinic again with a persistent need for an orthosis due to hip instability. The patient was prescribed physical therapy, and a magnetic resonance imaging scan in June 2022 revealed fatty degeneration of the gluteus superior and inferior muscles as well as the tensor fascia latae muscle.

The patient was then referred to our institution and presented in the neurosurgical outpatient clinic in October 2022. Physical examination revealed a weakness of hip abduction, atrophy of the right gluteal muscles, Trendelenburg sign, and a corresponding limp, which was counterbalanced by the patient by compressing the right greater trochanter with his ipsilateral hand. The neurology department was consulted to perform additional diagnostic and electroneurographic evaluations in October 2022 and confirmed a chronic lesion of the right superior gluteal nerve with completed reinnervation and a lack of pathological spontaneous activity in the right gluteus medius muscle. These findings excluded a further neurosurgical approach to restore the function of the gluteus medius muscle by means of nerve reconstruction or nerve transfer surgery. Therefore, the patient’s case was presented to our institution’s interdisciplinary board for peripheral nerve trauma in November 2022. The board recommended a salvage procedure; that is, shifting of the right vastus lateralis muscle to restore hip abduction. In accordance with the interdisciplinary board’s recommendation, the patient presented to our department’s outpatient clinic in January 2023. He still needed an orthosis for longer walking distances, although he was able to climb stairs without any problems. [See Video 1 [online], which displays preoperative gait. Note the marked hip abductor deficiency on the right side with a recognizable Trendelenburg sign.] After obtaining informed consent, we scheduled the abovementioned surgical procedure for May 2023.

**Video 1.** displays preoperative gait. Note the marked hip abductor deficiency on the right side with a recognizable Trendelenburg sign.

## SURGICAL TECHNIQUE

**Fig. 1.**
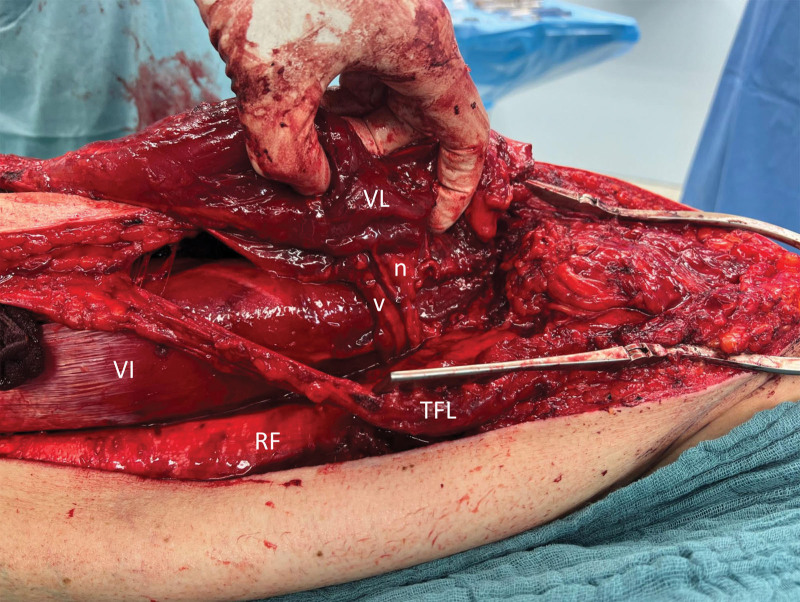
Dissection of the vastus lateralis muscle (VL) and its neuro(n)vascular(v) pedicle. VL, vastus lateralis muscle; VI, vastus intermedius muscle; RF, rectus femoris muscle; TFL, tensor fasciae latae muscle.

**Fig. 2.**
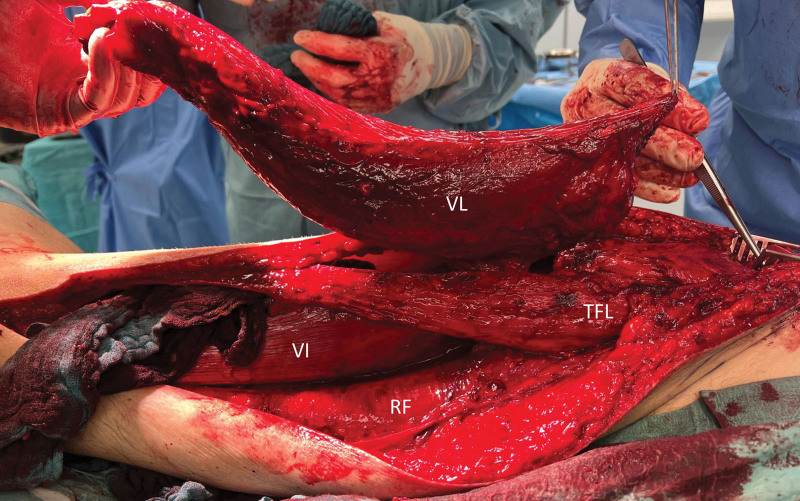
The vastus lateralis muscle is raised. VL, vastus lateralis muscle; VI, vastus intermedius muscle; RF, rectus femoris muscle; TFL, tensor fasciae latae muscle.

The patient was positioned supine and a wedge pillow was placed beneath the right hip. A lateral incision, extending from above the right greater trochanter to the lateral margin of the right patella, was performed to expose the tensor fasciae latae muscle and the iliotibial band. The iliotibial band was incised longitudinally, and the entire vastus lateralis muscle was carefully freed from the surrounding tissue. Subsequently, the muscle’s neurovascular pedicle in the proximal region was meticulously dissected (Fig. 1), and the muscle’s function was assessed using intraoperative nerve stimulation. The muscle was then mobilized from proximal to distal, and its attachment to the quadriceps tendon was separated (Fig. 2). [See Video 2 (online), in which the vastus lateralis muscle is raised and the new attachment sites are demonstrated.]

**Video 2:** in which the vastus lateralis muscle is raised and the new attachment sites are demonstrated.

**Fig. 3.**
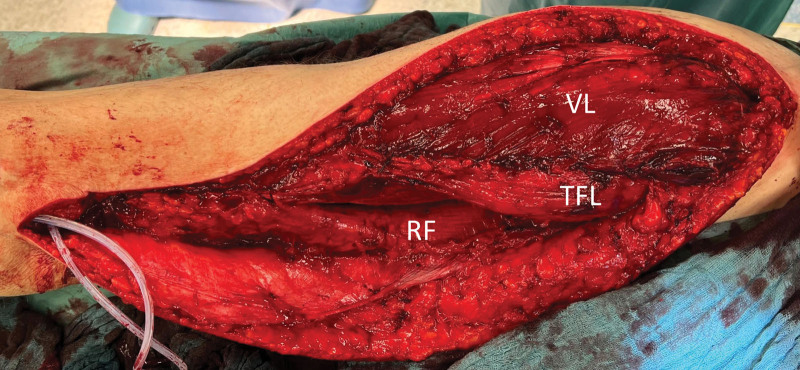
Operative site at the end of surgery. The vastus lateralis is proximally fixed to the ilium and greater trochanter while the muscle’s distal portion is sutured to a more proximal part of the quadriceps muscle. VL, vastus lateralis muscle; RF, rectus femoris muscle; TFL, tensor fasciae latae muscle.

**Fig. 4.**
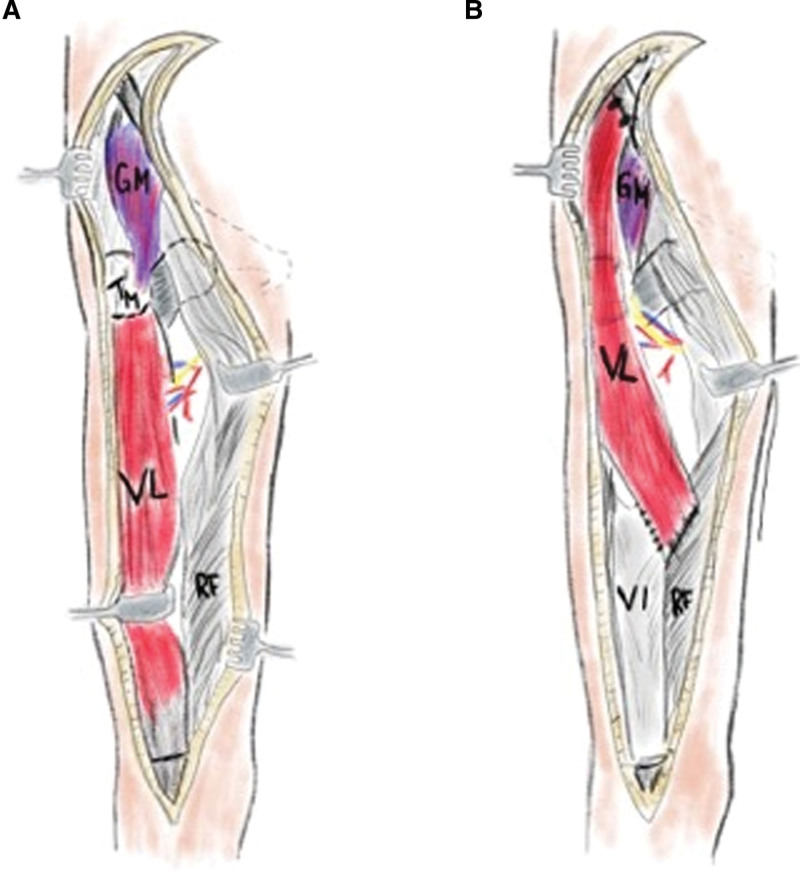
Schematic drawing of the operative site before and at the end of surgery. After detaching the vastus lateralis muscle’s proximal and distal parts (A) the muscle is reattached to the ilium and greater trochanter proximally and the rectus femoris muscle distally (B). GM, gluteus medius muscle; TM, greater trochanter; VL, vastus lateralis muscle; VI, vastus intermedius muscle; RF, rectus femoris muscle.

Dissection of the vastus intermedius muscle was performed with utmost care to preserve its nerve supply. The proximal section of the vastus lateralis muscle was then fixed to the ilium and greater trochanter using three 5-0 titanium anchor sutures. The muscle’s distal portion was sutured to a more proximal part of the quadriceps muscle using nonabsorbable multifile suture material (Figs. 3 and 4). An abduction splint was fashioned in a customized manner, and the patient was advised to wear it both day and night for a duration of six weeks postoperatively.

Six days postoperatively, the patient started mobilization and physical therapy with a knee brace and partial weight-bearing. Full weight-bearing was allowed 8 days postoperatively. The patient was discharged after 10 days and was able to ambulate with crutches. When the patient presented to the outpatient clinic 10 weeks postoperatively, he was in good overall condition without any relevant pain and demonstrated a significantly improved gait without the need for an orthosis. [See Video 3 [online], which displays the postoperative gait. Due to the vastus lateralis transfer, the patient’s gait has significantly improved and his limp has almost completely resolved. Additionally, note the marked excursion of the right patella during walking, which can be attributed to activation of the vastus lateralis muscle to enable hip abduction.]

**Video 3:** which displays the postoperative gait. Due to the vastus lateralis transfer, the patient’s gait has significantly improved and his limp has almost completely resolved. Additionally, note the marked excursion of the right patella during walking, which can be attributed to activation of the vastus lateralis muscle to enable hip abduction.

Physical examination revealed slight numbness in the gluteal area but normal function of the hip and knee joints. Trendelenburg sign was absent, but the patient occasionally required an orthosis for long distances due to a lack of muscle stamina.

## DISCUSSION

A transfer of the vastus lateralis muscle to reconstruct gluteus medius muscle function has mainly been reported in cases of hip abductor deficiency due to direct muscular injury so far.^9^ Several authors reported significant improvements following this procedure regarding pain, the range of movement, and walking ability, although it has to be noted that the overall number of patients was only 11 and four, respectively.^2,3^ Harvest of the vastus lateralis muscle is considered to cause only minimal functional deficits^10^ and no complications have been reported in the literature following the procedure described in this report,^3^ although the authors of a cadaveric study emphasized the imminent risk of damaging the motor branches to both the vastus intermedius and lateralis muscle when the latter is shifted too extensively.^4^ Therefore, the surgical technique can be demanding because the vastus lateralis is usually innervated in a two-fold manner with a longer distal and many short proximal branches, which are interwoven with branches of the lateral femoral cutaneus nerve. This limits the maximum range in which the muscle can be shifted to approximately 45 mm in the anterior and posterior direction, respectively. However, use of the aforementioned technique to restore hip abductor function following neurogenic injury has only been reported in two cases without illustrative media.^11^ We additionally modified the technique by anchoring the proximal part of the vastus lateralis muscle to the greater trochanter, thus mimicking the original line of pull of the gluteus medius muscle. The use of anchor sutures has been proven as a feasible option for tendon reattachment.^12^ Although transosseous sutures have been reported as stronger in comparison, anchor sutures provide sufficient strength for reattachment of the thigh muscles^13^ while also reducing the invasiveness of such procedures.^14^ In conclusion, we deem the vastus lateralis transfer as described in this case report a valuable technique to reconstruct hip abduction in patients in whom neurosurgical options are unavailable.

## DISCLOSURE

The authors have no financial interest to declare in relation to the content of this article.

## ACKNOWLEDGMENTS

We acknowledge support by Open Access Publishing Fund of University of Tübingen. This work was conducted in accordance with the ethical standards of the institutional and/or national research committee and with the 1964 Declaration of Helsinki and its later amendments or comparable ethical standards.
